# POLYMORPHISM OF THE COX-2 GENE AND SUSCEPTIBILITY TO COLON AND RECTAL CANCER

**DOI:** 10.1590/0102-6720201700020008

**Published:** 2017

**Authors:** Denise Camilios COSSIOLO, Helen Caroline Magalhães COSTA, Karen Barros Parron FERNANDES, Lino Luis Sanches LARANJEIRA, Marcos Tadeu Parron FERNANDES, Regina Célia POLI-FREDERICO

**Affiliations:** 1 Catholic University of Paraná, School of Medicine, Londrina, PR;; 2 Colorectal Surgery, Institute of Cancer of Londrina, Londrina, PR;; 3 UNOPAR, Rehabilitation Sciences Center, Londrina, PR, Brazil

**Keywords:** Colonic neoplasms, Rectal neoplasms, Gene frequency

## Abstract

**Background:**

The colorectal neoplasm is the fourth most common malignancy among males and the third among females. In the Western world is estimated that 5% of the population will develop it, making this disease a major public health problem.

**Aim:**

To analyze the prevalence of the polymorphism -765G / C region of the COX-2 gene in colorectal cancer patients compared to a control group, analyzing the possible association between this polymorphism and susceptibility to colorectal cancer**.**

**Method:**

This is a case-control study with 85 participants. Were selected 25 with colorectal cancer (case group) and 60 participants without colorectal neoplasia (control group). The molecular genetic analysis was perform to identify the polymorphism -765G / C COX2 gene with standard literature technique. In addition, patient’s clinical and pathological data were analyzed.

**Results:**

There was a light increase in prevalence between men in the case group, although this difference was not statistically significant. The results showed a high prevalence of GC and CC genotype in individuals with colorectal cancer, demonstrating an association between the presence of the polymorphism in the COX2 gene and susceptibility to colorectal cancer in this pattern (p=0.02). Similarly, there was also difference in allele frequencies in the groups. When patients with cancer were separated by tumor location, there was a higher prevalence of polymorphism in the left colon (p=0.02).

**Conclusion:**

The polymorphism in the COX2 gene is associated with increased susceptibility to colorectal cancer, specially rectosigmoid tumors.

## RESUMO

**Racional:**

A neoplasia colorretal representa a quarta malignidade mais comum entre homens e a terceira entre as mulheres. No mundo ocidental estima-se que 5% da população a desenvolverá, tornando-a grave problema de saúde pública.

**Objetivo:**

Analisar a prevalência de polimorfismo na região -765G/C do gene COX-2 em pacientes com câncer colorretal em relação a um grupo controle, analisando a possível associação entre este polimorfismo e a suscetibilidade a ele.

**Método:**

Foram incluídos neste estudo caso-controle 85 participantes. Selecionou-se 25 com neoplasia colorretal (grupo caso) e 60 pacientes sem neoplasia colorretal (grupo controle). Realizou-se análise genético-molecular para identificação do polimorfismo -765G/C do gene COX2 com técnica padrão da literatura. Além disso, foram levantados dados clínicos e anatomopatológicos dos pacientes.

**Resultado:**

Constatou-se discreto aumento de prevalência entre os homens no grupo caso, embora esta diferença não fosse estatisticamente significante. Os resultados revelaram alta prevalência do genótipo GC e CC nos indivíduos com câncer colorretal, demonstrando associação entre a presença do polimorfismo no gene COX2 e a suscetibilidade ao câncer colorretal nesta amostra (p=0,02). Similarmente, também se observou diferença nas frequências alélicas em relação aos grupos. Quando os pacientes com a neoplasia foram separados por localização do tumor, verificou-se maior prevalência do polimorfismo em pacientes de cólon esquerdo (p=0,02).

**Conclusão:**

O polimorfismo no gene COX2 está associado com a maior suscetibilidade ao câncer colorretal, especialmente em tumores do retossigmoide.

## INTRODUCTION

The colorectal neoplasm is one of the most common malignancies diseases in the world, being the fourth among male and the third in female, revealing to be an important public health problem^25^. Environmental and dietary factors influence the genesis of colorectal cancer (CRC). It is estimated that approximately 25% of all colorectal tumors have a family history^10^. However, there are still gaps in the identification of the early markers that would allow patients accompaniment and prognosis.

It is believed that prostaglandins are involved in the genesis of CRC. Prostaglandins are small molecules derived from arachidonic acid, which are produced from the enzyme cyclooxygenase (COX). This enzyme is subdivided into COX-1, responsible for physiological activities and therefore is called constitutive, and COX-2 expressed after stimulation of cytokines, growth factor and mitogens^1^.

In physiological conditions, COX-2 is expressed in tissue of the gastrointestinal tract at very low levels. However, there is an increase of its expression in CRC^21^. Thus, a study by Eberhart et al verified that approximately 85% of colon and rectal tumors have elevated levels of COX-2^8^. Reports in the literature suggest that single nucleotide polymorphism (SNP) in the COX-2 gene would be able to modify enzyme function and, therefore, to expand the risk of an individual to develop colon cancer^24^.

 The changes in the COX-2 gene may imply changes in enzyme function and contribute to the inflammatory response or to the increase risk of developing CRC^5^ ^,^ ^15^. However, even though several polymorphisms have already been identified in the COX-2 gene, no conclusive relation between this gene and the genesis of CRC has been established^21^. In this way, there is the possibility to establish the expression of COX-2 as a prognostic factor, since it has been linked to the development of metastases and with a lower survival rate^8^.

In this context, the present study aims to estimate the allele and genotype frequencies of the COX-2 gene polymorphism, in order to evaluate the possible association between these frequencies and the susceptibility to CRC.

## METHODS

This study was reviewed and approved by the Research Ethics Committee (Protocol no. 884/397) of the Institution. This was a case-control study, conducted in partnership with the Institute of Cancer of Londrina. All patients were previously informed about the objectives and procedures to be performed and had signed the Informed Consent Term.

### Patients

The patients included had CRC with surgical indication. Those who lost follow-up shortly after surgery were excluded. For the control group, individuals without colorectal neoplasm and matched by gender and age group were included in relation to the case group. On the other hand, individuals with a history of other neoplasms or with the presence of polyps during colonoscopy were excluded from the sample.

### Clinical data

From the clinical information of the patients, demographic and anthropometric data were extracted: gender, age, BMI, history of smoking and alcoholism. Blood analysis provided information on lymphocytometry, hemoglobin, calcium, transferrin, albumin, initial and post-operative carcinoembryonic antigen, from lymphocyte copy (absolute) of the leukogram both at admission and at discharge. In addition, it was evaluated the results of the anatomopathological study and staging of the tumor provided by the pathological report and/or the oncologist. Pre-operative rectosigmoidoscopies/colonoscopies were analyzed, containing the description of the size of the preoperative lesion and the site of the tumor. Also, it was taken into consideration the treatments and operations made, the description of the procedure if it was performed and if it was necessary second or third intervention. In order to obtain information about death and survival, telephone contact with patient or family was used to identify the patient’s current situation.

### Blood collection

Was performed in peripheral vein in vacuum tubes containing anticoagulant (EDTA). The tubes were centrifuged and the nucleated cells (leukocytes) were separated for further DNA extraction or stored in freezer storage at -80° C until the time of extraction. The patterns were transferred to sterile flasks and DNA was obtained after enzymatic digestion in buffer containing proteinase K and SDS and subsequent extraction with organic solvents (phenol/chloroform).

### Molecular analysis of the COX-2 gene polymorphism

After DNA extraction from the samples, the analysis of the polymorphism -765G / C region of the COX-2 gene was evaluated by the technique of PCR of restriction fragment length polymorphism (PCR-RFLP), being the primer for PCR selected according to the genome database (region sequence -765G / C: Forward” 5’-ATT CTG GCC ATC GCC GCT TC-3’ and “Reverse”, 5’-CTC CTT CTT TCT TGG AAA GAG CG-3’). Amplification conditions are determined by these conditions of temperatures and times: 94° C - 3 min; 94° C, 59° C and 72° C for 60 s each, with 35 cycles and 72° C for 5 min. For the proline allele detection, the parameters will be used: 94° C - 3 min; 94° C, 57° C and 72° C for 30 s each, with 35 cycles and 72° C for 5 min. After PCR the products were subjected to cleavage with the restriction enzyme called BstUI (Bsh 1236I) for 8 h at 37° C. The amplified products were subjected to separation by electrophoresis and 2% agarose gel and were stained by Sybr Safe. Amplified fragments of 134 and 23 base pairs (bp) will indicate homozygous for the wild-type allele (-765GG). A single fragment of 157 base pair will indicate homozygosis for the C allele (-765CC) and the presence of three fragment of 157, 134 and 23 base pair will indicate heterozygosis (-765GC).

## RESULTS

This study had 85 participants. Were selected 25 of them with CRC (age 56.3±12.2 years) and 60 participants of the control group (age 60.5±6.8 years ). Regarding gender, there was a slightly increase on the prevalence of men in the case group (n=14, 56.0%), although this difference was not statistically significant.

There was a high prevalence of GC and CC genotype in individuals with CRC, noting the association between the presence of COX2 gene polymorphism and susceptibility to CRC in this sample (Fisher-Freeman-Halton test, p=0.02, Table 1). There were also differences in the allelic frequencies in relation to the groups (Chi-square test, p <0.05, Table 2).

**TABLE 1:** Genotypic frequencies of the COX2 gene in relation to colorectal cancer

Genotypicfrequencies	Colorectal cancer		Total
	No	Yes	
GG	43	11	54
	71.7%	44.0%	63.5%
GC	16	13	29
	26.7%	52.0%	34.1%
CC	1	1	2
	1.7%	4.0%	2.4%
Total	60	25	85
	100.0%	100.0%	100.0%

**TABLE 2:** Distribution of the allelic frequencies of the COX2 gene and in individuals with and without colorectal cancer

Allelic frequencies	Colorectal cancer		Total
	No	Yes	
G	102	35	137
	85.0%	70.0%	80.60%
C	18	15	33
	15.0%	30.0%	19.40%
Total	120	50	170
	100.0%	100.0%	100.0%

When the genotype frequencies of the gene in question were analyzed in relation to the presence and location of the tumor, there was prevalence of GC and CC genotype in patients with cancer and whose location was in the left colon. This confirms that there is a more significant association between the presence of the COX-2 gene polymorphism with tumors located in the left colon (Fisher-Freeman-Halton test, p<0.05, Table 3).

**TABLE 3:** Distribution of the genotypic frequencies of the COX2 gene in relation to the presence and location of the tumor

Genotypic frequencies	Colorectal cancer		Control	Total
	Right colon	Left colon		
GG	7	4	43	54
	70.0%	26.7%	71.7%	63.5%
GC	3	10	16	29
	30.0%	66.7%	26.7%	34.1%
CC	-------	1	1	2
	-------	6.7%	1.7%	2.4%
Total	10	15	60	85
	100.0%	100.0%	100.0%	100.0%

## DISCUSSION

The enzyme COX-2 is responsible for the conversion of arachidonic acid to prostaglandin. Its action is inducible by numerous mediators present in the inflammatory process. Studies have shown a significant increase of the expression of COX-2 in several types of neoplasm, especially the epithelial ones, including the CRC^3^ ^,^ ^9^. The single nucleotide polymorphism with the exchange of a guanine by a cytosine at the -765 position of the COX2 gene has been described as a risk factor for the occurrence of CRC^12^.

The results of this study showed an association of the polymorphic C allele with increased susceptibility to CRC. When comparing the patients who presented the neoplasm (case group) with the control group, the GC and CC genotypes prevailed in the individuals carrying the CRC. This association was also preserved by subdividing the neoplasm group according to the site of the occurrence of the primary tumor in the right colon and left colon; however, was more strongly significant in lesions from the left colon. These results are consistent with data from some of previous studies, including the meta-analysis of Cao et al and Peng et al, which analyzed 6774 cases of CRC and 9772 controls^19^ ^,^ ^30^. Despite this, some studies have shown no correlation between the polymorphism -765G / C region of the COX-2 gene and colorectal neoplasms^7^ ^,^ ^24^ ^,^ ^27^. Analysis of all these studies demonstrates predisposition to correlate single nucleotide polymorphism and CRC in Asian populations, but this does not apply to Caucasian populations.

There is hypothesized that the COX-2 enzyme is involved in the stimulation of cell proliferation and angiogenesis, in the inhibition of apoptosis and in immunological suppression, all events with carcinogenic potential ^11^ ^,^ ^14^ ^,^ ^17^ ^,^ ^29^. The present study analyzed single nucleotide polymorphism with the exchange of a guanine with a cytosine at position -765 of the COX2 gene^18^. This polymorphism occurs in the promoter region of the gene, resulting in a possible increase in gene expression with consequent elevation of levels of the COX-2 protein^26^. In this context, the study by Eberhart et al. demonstrated that COX-2 levels were elevated in 85% of CRC cases^8^.

There are many evidence of the influence of the polymorphism -765 G / C region on the genesis of several tumors, such as prostate, breast, ovary, lung, liver cancer and CRC^1^ ^,^ ^4^ ^,^ ^13^. The association found in the current study was for the occurrence of CRC at any location, but it was more significant in cases of left colon tumors than in right colon tumors. This reinforces the hypothesis that, although they are in the colon, they are neoplasms of different causes, as suggested by Peng et al. in his meta-analysis. In the rectosigmoid, lesions have the appearance and behavior more predominantly inflammatory, which would be a possible explanation for this higher correlation of COX-2 with the tumors on the left compared to the cases of right colon.

In Brazil, COX-2 polymorphism studies and the occurrence of colorectal tumors are scarce, and this study is one of the first reports comparing not only the susceptibility to cancer but also the association with tumor topography. In this context, this study contributes to the characterization of the association of the polymorphism in the COX2 gene with the CRC in the Brazilian population, notably more significant in the retossigmoid lesions. 

Polymorphism research can provide significant future advances, as it will enable the identification of the individuals most susceptible to the risk of tumors, allowing individualization in the therapeutics. Regarding colorectal neoplasm, polymorphic patients with the C allele were potential candidates to the treatment with anti-inflammatory drugs, especially in cases of left colon cancer.

## CONCLUSION

Polymorphism in the COX-2 gene is associated with increased susceptibility to colorectal cancer, especially in tumors of the rectosigmoid segment.
